# Minute Subdivision of Coloring Matter

**Published:** 1877-01

**Authors:** 


### MINUTE SUBDIVISION OF COLORING MATTER.

The remarkably minute quantity of dye-stuff required to tint an otherwise colorless liquid, such as water or alcohol, is well shown by Herr Annaheim in a paper presented to the Berlin Chemical Society. He dissolves .0007 gramme of fuchsine in alcohol, and dilutes the solution to 1000 centimetres, so that each centimetre contains .0000007 grammes of fuchsine, an amount of color which appears quite strong when backed by white paper. By still further experiments he shows that .00000002 gramme of fuchsine is perceptible as color by the human eye, and by calculation that the absoute weight of an atom of hydrogen in a molecule of the dye can not exceed .000000000059 gramme.

